# External quality assessment of molecular detection and variant typing of SARS-CoV-2 in European expert laboratories in 2023

**DOI:** 10.1128/jcm.01538-24

**Published:** 2025-03-14

**Authors:** Kim C. Heimsch, Kamelia R. Stanoeva, Ramona Mögling, Annette Kraus, Eeva K. Broberg, Jan Felix Drexler, Chantal B. E. M. Reusken, Adam Meijer, Christian Drosten, Victor M. Corman

**Affiliations:** 1 Institute of Virology, Charité-Universitätsmedizin Berlin, corporate member of Freie Universität Berlin and Humbolt-Universität zu Berlin 111617 , Berlin, Germany; 2 Centre for Infectious Disease Control, National Institute for Public Health and the Environment (RIVM) 571105 , Bilthoven, the Netherlands; 3 European Centre for Disease Prevention and Control (ECDC) 56756 , Stockholm, Sweden; 4 German Centre for Infection Research (DZIF), associated partner site Charité 459706 , Berlin, Germany; 5 Labor Berlin-Charité Vivantes 646268 , Berlin, Germany; Wadsworth Center - NYSDOH, Albany, New York, USA

**Keywords:** SARS-CoV-2, variants, COVID-19, external quality assessment, molecular diagnostics, Europe

## Abstract

The COVID-19 pandemic highlighted the importance of laboratory preparedness. Regular monitoring of diagnostic tools via external quality assessments (EQAs) is key to maintaining robust public health response service. We hereby conducted a third SARS-CoV-2 EQA assessing the diagnostic capabilities of European expert public health laboratories. A 10 samples panel containing Alpha (used in previous EQA), BA.4, BA.5, and BQ.1.18 variants along with human seasonal coronaviruses and negative controls was produced and validated. Participants were invited by the European Centre for Disease Prevention and Control (ECDC) and asked to submit results and assay details via electronic forms. Thirty-eight laboratories from 31 European countries participated. Most (*n* = 32, 84%) identified all panel samples correctly and used in-house (11, 29%), commercial assays (22, 58%), or both (5, 13%). Compared to previous EQAs, correct detection of the SARS-CoV-2 samples in the panels increased: 8 (12%) in 2020, 45 (75%) in 2021, and 34 (90%) laboratories in 2023, respectively. The number of participants decreased to an average of one laboratory per country (range 1–3) compared to two (1–7) laboratories in both previous EQAs. The usage of commercial assays gradually increased in contrast to the usage of in-house or both approaches. The capacity for SARS-CoV-2 molecular diagnostics has markedly improved in Europe as evidenced by three consecutive EQAs carried out by expert public health laboratories. Routine monitoring of diagnostic and surveillance assays via EQAs remains key to maintaining rapid public health laboratory response systems.

**IMPORTANCE:**

External quality assessments (EQAs) are crucial to ensure the reliability and consistency of diagnostic laboratories. They provide an objective framework for evaluating the performance of testing systems, enabling laboratories to identify weaknesses and implement improvements promptly. In the context of SARS-CoV-2, EQAs have become even more critical due to the high demand for accurate molecular diagnostics and the emergence of new variants. Accurate detection and typing of variants are especially essential for monitoring viral evolution. EQAs help standardize methodologies, ensuring that results across laboratories remain comparable and trustworthy. Moreover, they play a pivotal role in minimizing errors such as false positives or negatives. In this rapidly evolving landscape, regular EQAs are indispensable for maintaining high-quality standards in molecular diagnostics and variant surveillance. We demonstrate here that regular EQAs improve the molecular detection of SARS-CoV-2 in European laboratories.

## INTRODUCTION

The severe acute respiratory syndrome coronavirus 2 (SARS-CoV-2) pandemic highlighted the importance of laboratory preparedness and maintaining robust diagnostic and response services. Regular monitoring, via conducting external quality assessments (EQAs), is key to maintaining capacity and capability to rapidly and correctly detect pathogens and monitor their evolution (variant monitoring). In Europe, two EQAs of molecular detection and characterization of SARS-CoV-2 that were organized in 2020 and 2021 (1, 2) showed improvement in diagnostic sensitivity but still revealed detection issues with samples with low RNA concentrations. Here, we report on a follow-up EQA to assess again the performance of European expert laboratories, including testing capacities for recent strains in the aftermath of the COVID-19 pandemic.

## MATERIALS AND METHODS

### Participants

The European Centre for Disease Prevention and Control (ECDC) outsourced the implementation of this EQA to the AURORAE consortium (3) and invited operational contact points for the European COVID-19 reference laboratory network (ECOVID-LabNet) to register by the end of March 2023. The aim was to include all national reference laboratories or laboratories acting as such involved in the COVID-19 response, similar to the two previous European SARS-CoV-2 EQAs. The list of participating laboratories is given in the Acknowledgements.

### Panel composition

The EQA panel contained 10 samples: seven with inactivated material of different SARS-CoV-2 lineages (Alpha variant B.1.1.7, and Omicron variants BA.4, BA.5, and BQ.1.18), two controls with inactivated material of two seasonal human coronaviruses (HCoV-229E and HCoV-OC43), and one negative control with phosphate-buffered saline (PBS) (Table 1). Two different concentrations of BA.4, BA.5, and BQ.1.18 were included in the panel to evaluate sensitivity. The lowest concentrations of BA.4 and BA.5 were 12 and 13 cps/µL, respectively (resuspended EQA vial). These variants were chosen to represent the circulating variants at the time the EQA was conducted (4). The Alpha (B.1.1.7) variant, in comparable concentration to the previous EQA (2), was included for consistency as it was part of the panel in the previous SARS-CoV-2 EQA (2). The panel was produced at Charité-Universitätsmedizin Berlin.

**TABLE 1:** EQA panel composition and performance of 38 laboratories participating in the European SARS-CoV-2 EQA, 2023^*a*^

Sample ID	Virus	Lineage	Mutations	Copies/µL	Correct molecular detection		Correct SARS-CoV-2 variant determination	
					%	No./total labs	%	No./total labs
CoV_ID_1015	SARS-CoV-2	B.1.1.7	H69/V70 del Y144 del N501Y A570D P681H T716I S982A D1118H	120	97	37/38	31	4/13
CoV_ID_1003	SARS-CoV-2	BA.4	T19I L24/P25/P26 del A27S H69/V70 del G142D V213G G339D S371F S373P S375F T376A D405N R408S K417N N440K L452R S477N T478K E484A F486V Q498R N501Y Y505H H655Y N679K P681H N764K D796Y Q954H N969K	12	95	36/38	27	3/13
CoV_ID_1008	SARS-CoV-2	BA.4	T19I L24/P25/P26 del A27S H69/V70 del G142D V213G G339D S371F S373P S375F T376A D405N R408S K417N N440K L452R S477N T478K E484A F486V Q498R N501Y Y505H H655Y N679K P681H N764K D796Y Q954H N969K	85	100	38/38	46	6/13
CoV_ID_1011	SARS-CoV-2	BA.5	T19I L24/P25/P26 del A27S H69/V70 del G142D V213G G339D S371F S373P S375F T376A D405N R408S K417N N440K L452R S477N T478K E484A F486V Q498R N501Y Y505H H655Y N679K P681H N764K D796Y Q954H N969K	13	95	36/38	8	1/113
CoV_ID_1005	SARS-CoV-2	BA.5	T19I L24/P25/P26 del A27S H69/V70 del G142D V213G G339D S371F S373P S375F T376A D405N R408S K417N N440K L452R S477N T478K E484A F486V Q498R N501Y Y505H H655Y N679K P681H N764K D796Y Q954H N969K	70	97	37/38	39	5/13
CoV_ID_1002	SARS-CoV-2	BQ.1.18	T19I L24/P25/P26 del A27S H69/V70 del G142D V213G G339D S371F S373P S375F T376A D405N R408S K417N N440K K444T L452R N460K S477N T478K E484A F486V Q493Q Q498R N501Y Y505H H655Y N679K P681H N764K D796Y Q954H N969K	160	100	38/38	46	6/13
CoV_ID_1019	SARS-CoV-2	BQ.1.18	T19I L24/P25/P26 del A27S H69/V70 del G142D V213G G339D S371F S373P S375F T376A D405N R408S K417N N440K K444T L452R N460K S477N T478K E484A F486V Q493Q Q498R N501Y Y505H H655Y N679K P681H N764K D796Y Q954H N969K	3E + 05	97	37/38	69	9/13
CoV_ID_1004	Human coronavirus 229E			225	100	38/38		
CoV_ID_1018	Human coronavirus OC43			98	95	36/38		
CoV_ID_1001	-				97	37/38		

^*a*^ Concentrations in cps/µL were determined by RT-PCR. For SARS-CoV-2-containing samples, an in-house assay targeting the E gene was used (5). Samples containing seasonal HCoVs were tested using specific in-house RT-PCR assays (6). The mutations listed are determined in comparison to SARS-CoV-2 B.1 as reference. -, no virus contained.

### Panel preparation, validation, and dispatch

All SARS-CoV-2 samples were isolated from clinical specimens using Vero E6 cells cultivated in Dulbecco’s modified Eagle medium (DMEM), supplemented with 2% fetal calf serum (FCS). HCoV-229E BN_1 (7) was stocked using HUH-7 cells cultured in DMEM, supplemented with 2% FCS. The HCoV-OC43 sample was isolated from a clinical specimen using human bronchial epithelial cells cultivated at the air-liquid interface (ALI) using the PneumaCult ALI medium supplemented with Hydrocortisone, Heparin, Primocin, and Penicillin and Streptomycin.

Inactivation of all samples was performed using a final concentration of 0.05% betapropiolactone (BPL). The virus/BPL mixture was incubated at 4°C for 14–16 h, followed by an incubation at 37°C for 2 h. To confirm successful inactivation of SARS-CoV-2, the samples were passaged three times consecutively in Vero E6 cells, cultured in DMEM supplemented with 2% FCS.

Inactivated samples were diluted to the desired concentration with PBS. Aliquots of 500 µL of each sample were lyophilized in screw-cap vials. The lyophilized samples were shipped to participants at ambient temperature.

All SARS-CoV-2 samples were quantified using the E-gene target in an RT-PCR-based in-house assay (5). Samples positive for HCoV-229E and HCoV-OC43 were quantified using specific RT-PCR-based in-house protocols (6). RT-PCR-based validation of samples was performed at three institutions, namely Charité-Universitätsmedizin Berlin, the National Institute for Public Health and the Environment of the Netherlands, and the Robert Koch Institute, Berlin, independently. The samples were additionally sequenced by one of the institutes. All specimens performed as expected by all three laboratories and were valid for use in the panel.

### Data collection

Results were reported via an online survey form (hosted on the EU survey platform). Participants were asked to score their samples (positive, negative, and inconclusive). Furthermore, participating laboratories were asked to give information about cycle threshold (ct) values, extraction method, and types of RT-PCRs (in-house protocol and/or commercial kit) for each target and each sample. In addition, participants could report the determined SARS-CoV-2 variant (by WHO label or Pango lineage) and/or signature mutations, together with information regarding sequencing methods used, if any. Identifying variants and their specific features was an optional activity in this EQA.

### Evaluation of results

Results of the molecular detection were scored according to the following system: correctly reported samples (presence or absence of SARS-CoV-2 RNA): 0 points, inconclusive results: 1 point, and false negative or positive results: 2 points. For sequencing results, we differentiated between correctly determined variants/signature mutations, general identification as variant of concern (VOC), and false typing. Providing the correct variant name, Pango classification, or signature mutation(s) were all considered equally correct.

In addition, we compared this EQA with previous ones with regard to the number of participants, EQA participation for the first time, number and type of methods used, and overall performance.

### Statistical analysis

Data cleaning and analysis were performed using R (version 4.3.0). Figures were prepared using GraphPad Prism (version 9.0) or R (version 4.3.0).

## RESULTS

### Participation

Thirty-eight expert laboratories from 31 countries, covering 26 of the 30 EU/EEA countries and 5 of the 7 EU pre-accession countries, reported EQA results (Fig. S1).

### Molecular detection of SARS-CoV-2

An overview of the results for the individual samples is shown in Table 1. Regarding the detection of SARS-CoV-2 RNA, 31 of 38 participating laboratories correctly classified all 10 samples, 11% (4/38) classified 9 samples correctly, and 8% (3/38) classified 8 or fewer samples correctly. Of all reported results, 3% (10/380) were incorrect. Among the SARS-CoV-2 containing samples, 1.1% (3/266) were reported as inconclusive and 1.5% (4/266) as negative. Three laboratories (7.9%; 3/38) showed inconclusive results for one or two samples. The three samples with the lowest concentrations (≤70 viral RNA copies [cps]/µL) were not reported as positive by one laboratory (2.6%; 1/38). Two laboratories had false positive results for one sample each (5.2%; 2/38), whereby one laboratory tested the negative control positive, and the other laboratory showed the OC43 containing samples as positive for SARS-CoV-2. Another laboratory indicated the OC43-containing sample as inconclusive.

**Fig 1:**
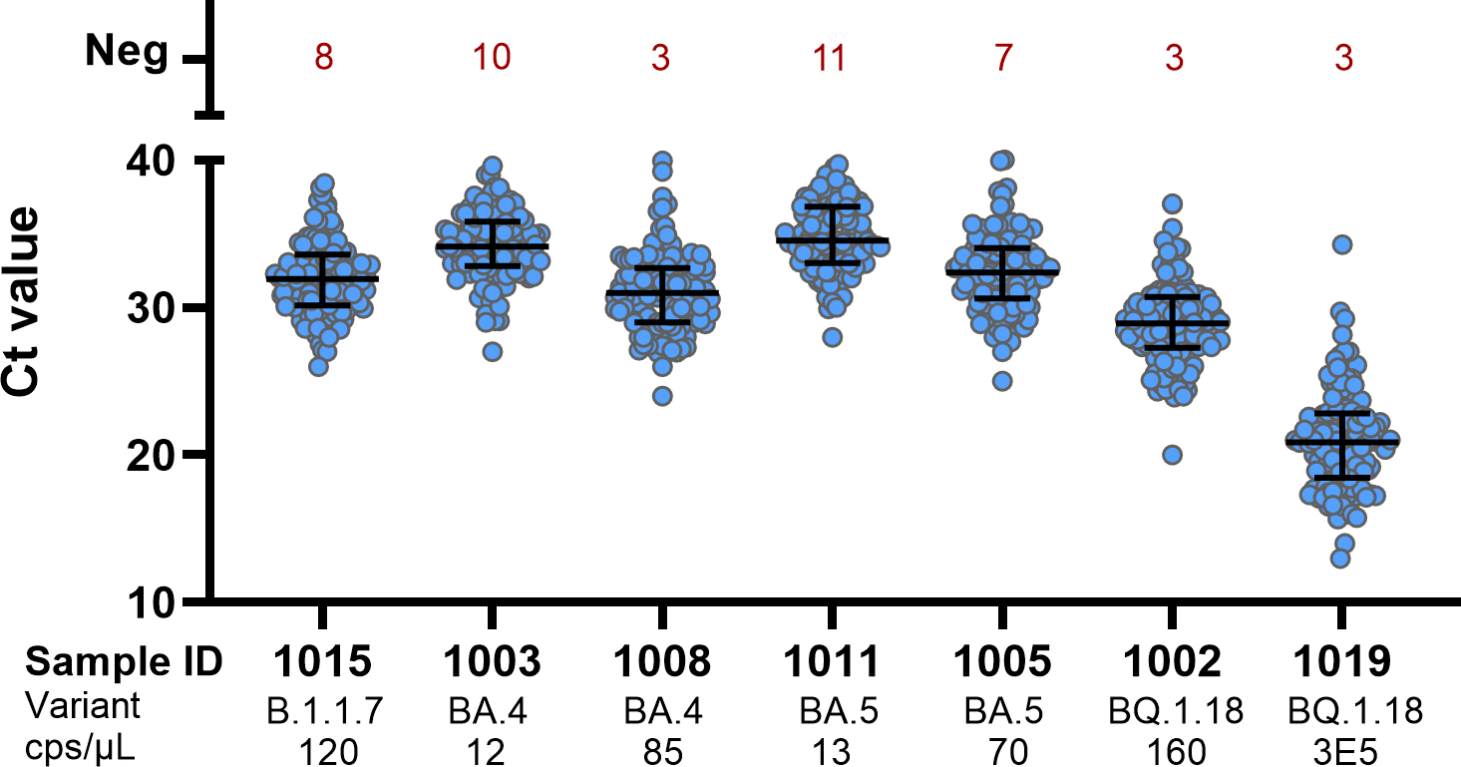
Ct values of SARS-CoV-2 containing samples. Multiple answers per laboratory are included. Shown are median ct values with interquartile range.

The ct values of positively tested samples were in general lower with increasing SARS-CoV-2 RNA concentration (Fig. 1). The detected ct values for the individual sample varied depending on the detection target and tests used.

### SARS-CoV-2 variant typing

**Fig 2:**
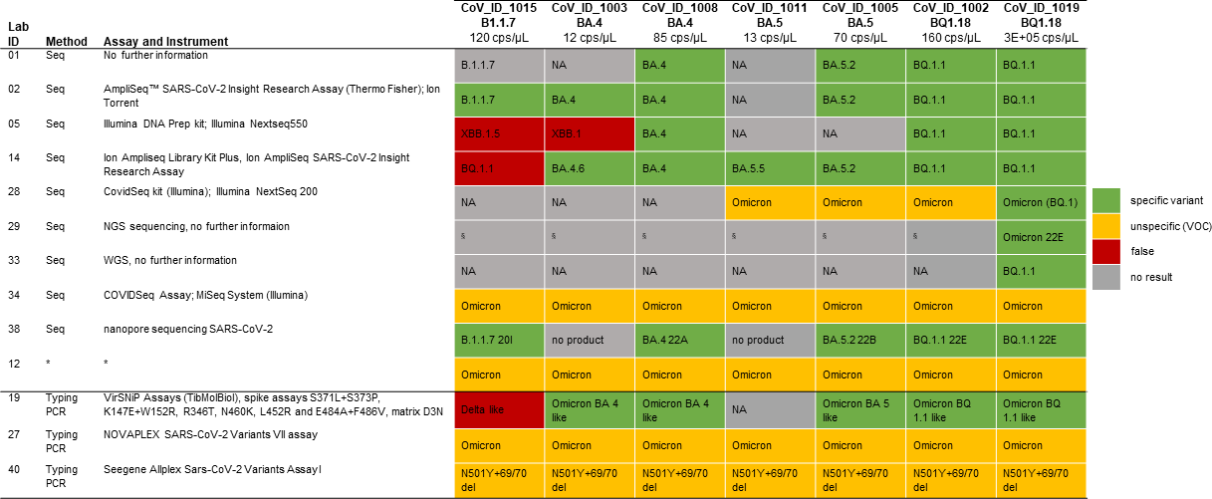
SARS-CoV-2 variant typing results and overview of typing methods of laboratories participating in the optional typing exercise, European SARS-CoV-2 EQA, 2023. VOC, variant of concern. Specific variant, the SARS-CoV-2 variant or specific mutations were named; unspecific, named as variant but not the specific sublineage; false, wrong VOC was named. *, no information given.

Variant typing was reported by 13 participating laboratories. Three laboratories used typing PCRs to identify the variant. Nine laboratories performed sequencing. One laboratory did not specify the method used. The quality of sequencing results was better for samples with higher concentrations of viral RNA (Fig. 2; Table 1). Nine (69%) of 13 laboratories that performed typing identified the highest concentration sample correctly. The samples with the second highest concentration were reported correctly by 46% (6/13). The sample containing B.1.1.7 was correctly identified by three laboratories (27%). Six laboratories (46%) identified this sample as false and reported Omicron variants or mentioned “delta-like” for this sample. For the two samples with the lowest concentrations (12 and 13 cps/µL), 27% (3/13) and 8% (1/13) of the laboratories determined the variant correctly. One laboratory reported all samples to be VOCs without further identifying the specific virus variant (Fig. 2). This laboratory performed typing PCR using the Seegene Allplex SARS-CoV-2 Variants Assay I kit. In addition, two laboratories stated that NGS failed because the quality of the sequences was too low for interpretation. This was mentioned for all SARS-CoV-2 containing samples. Low quality of sequences was also the reason why some laboratories stated “no result” for some samples. For many samples, it was not further defined why typing failed. Different kits were used for sequencing, like the Ion AmpliSeq SARS-CoV-2 Insight Research Assay (Thermo Fisher), the DNA prep kit from Illumina, or the CovidSeq kit from Illumina.

Figure 2 provides an overview of used typing methods and the results of the lineage determination per sample by each laboratory.

### Comparison with two previous European SARS-CoV-2 EQAs

The number of participating laboratories decreased over time from 68 participants in the first EQA, conducted in 2020, to 38 participants in this EQA. The same institutes were not consistently invited to the EQAs. There was an average of one participating laboratory per country, as compared to two in the previous EQAs (Table 2). In this EQA, 31 countries were represented in comparison to 35 and 34 in the first two EQAs. All participants of the current EQA also participated in the first and/or second EQAs.

**TABLE 2:** Comparison of three SARS-CoV-2 European EQAs^*a*^

	1st EQA 2020	2nd EQA 2021	3rd EQA 2023
Participation			
Participating laboratories (*n*)	68	60	38
Represented countries (*n*)	35	34	31
Represented EU/EEA countries (*n*)	28	28	27
Represented pre-accession countries (*n*)	5	6	4
Participating laboratories per country (average; min–max)	2; 1–7	2; 1–7	1; 1–3
Methods (*n* of laboratories; % of all participants)			
Automated extraction	50; 74%	43; 72%	30; 79%
Manual extraction	18; 26%	17; 28%	8; 21%
Only in-house methods used	21; 31%	12; 20%	11; 29%
Only commercial methods	27; 40%	33; 55%	22; 58%
Both in-house and commercial methods	20; 29%	15; 25%	5; 13%
Determination of virus variants by PCR or sequencing	NA^*d*^	37; 60%	15; 39%
Sequencing performed	2; 3%^*b*^	14; 23%	7; 18%
Results (*n* of laboratories; % of participants)			
All SARS-CoV-2 containing samples correctly classified	8; 12%	45; 75%	34; 90%
>50% of SARS-CoV-2 containing samples correctly classified	23; 34%	60; 100%	38; 100%
All no SARS-CoV-2 containing control samples correctly classified	37; 54%	59; 98%	35; 92%
All SARS-CoV-2 containing samples with variants and/or mutations identified (*n* of laboratories; % of laboratories identifying variants)^*c*^	NA	3; 8%	1; 7%
>50% of SARS-CoV-2 containing samples with variants and/or mutations identified (*n* of laboratories; % of laboratories identifying variants)^*c*^	NA	26; 70%	10; 67%

^*a*^ Differences in participation, differences in methods, and differences in results are shown.

^*b*^ The first EQA did not include determination of SARS-CoV-2 variants, and use of sequencing could only be mentioned as a free-text comment. Thus, the number here might not represent the actual use of sequencing methods.

^*c*^ Includes either the identification of the specific SARS-CoV-2 variant and/or mutations, or a correct though unspecific characterisation, for example, variant of concern.

^*d*^ NA, not applicable.

**Fig 3:**
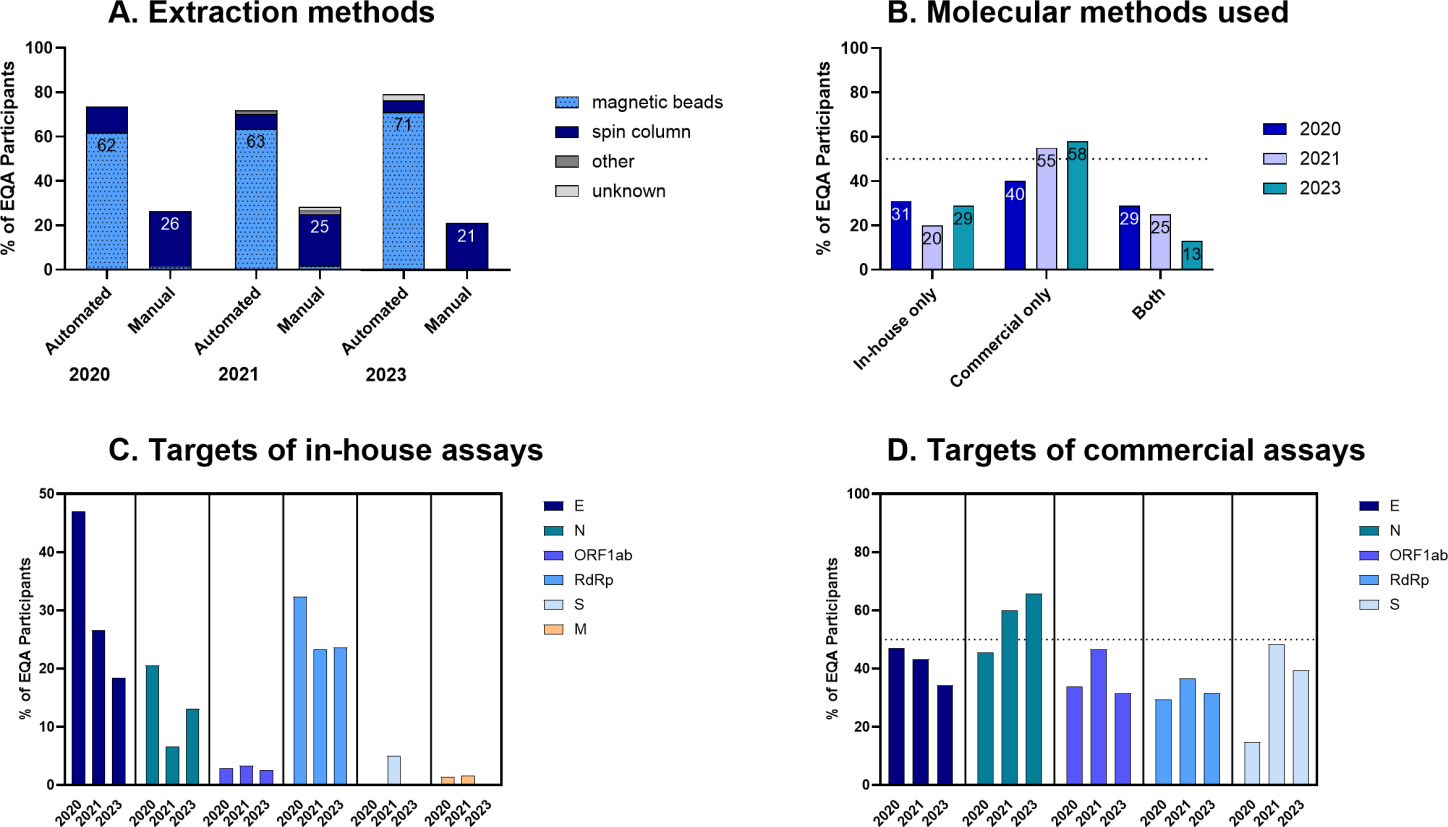
Comparison of three EQAs in terms of (**A**) extraction methods used, (**B**) molecular methods used, (**C**) targets detected using in-house assays, and (**D**) targets detected using commercial assays. Percentage of participating laboratories per corresponding EQA year is visualized.

In line with increasingly streamlined diagnostics, a high number of laboratories used automated extraction over the course of the three EQAs (79% in the recent EQA). Automated extraction was predominantly done with a magnetic beads-based method, whereas manual extraction was almost entirely spin column-based (Fig. 3A). In all three EQA rounds, a higher number of participating laboratories used commercial (as opposed to in-house) methods for molecular diagnostics (Fig. 3B). The proportion of laboratories using only commercial methods for SARS-CoV-2 detection increased from 40% in 2020 to 55% in 2021, reaching 58% in the current EQA round. Conversely, laboratories using both in-house and commercial methods decreased to 13% in 2023, compared to 29% in 2020 and 25% in 2021, amounting to 71% of participants in 2023 who used commercial assays (Table 2; Fig. 3B). Laboratories used and reported on average a total of two different molecular methods (in-house and/or commercial) in each EQA, ranging from one to five in 2020 and one to four assays in 2021 and 2023, respectively. The targets for in-house assays were predominantly E gene and RdRp (Fig. 3C), whereas targets of commercial assays varied wider between E, N, ORF1ab, RdRp, and S (Fig. 3D). For this EQA, the target detected mostly via in-house assays was RdRp (23.7%) and via commercial assays N (65.8%) (Fig. 3C and D).

**Fig 4:**
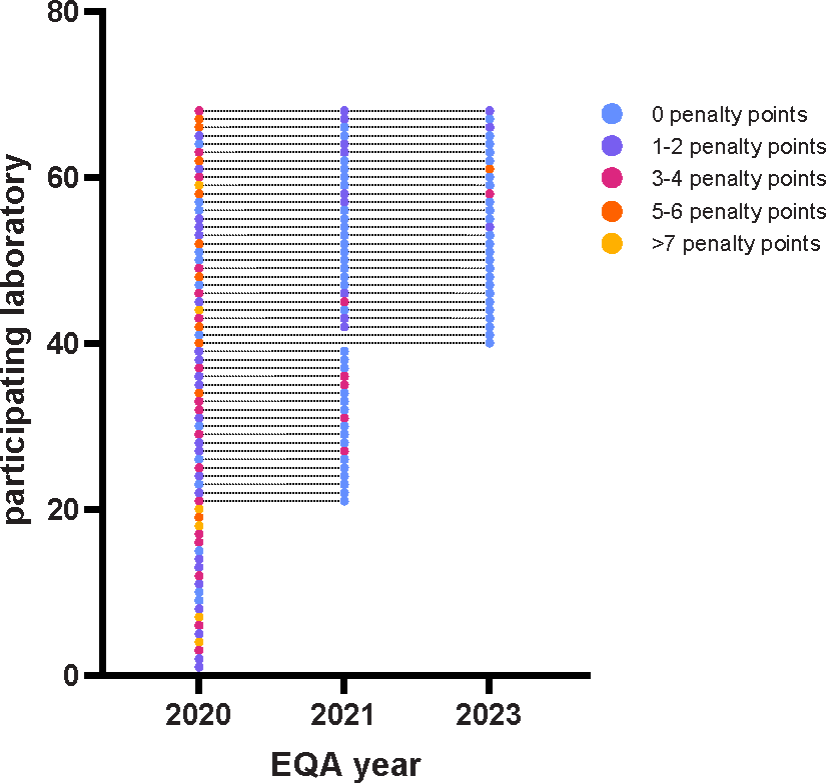
Participating laboratories and their penalty points for three European SARS-CoV-2 EQAs. Laboratories participating in multiple EQAs are connected via dashed lines.

An overall improvement in SARS-CoV-2 detection was observed over the course of the three EQAs (Table 2; Fig. 4). The percentage of laboratories correctly identifying all samples increased from 12% in 2020 to 75% in 2021, reaching 90% for the current EQA (Table 2). The detection of the variant that was included in the second and this EQA (B.1.1.7) was comparable between the 2021 and the current EQA (72.9–100%; 97%). In 2021, four B.1.1.7 containing samples with concentrations of 2.5, 3, 11, and 290 cps/µL were included in the panel. In the current EQA, one B.1.1.7 containing sample with 120 cps/µL was part of the panel. Other variants of the panel represented the variants circulating at the time of the respective EQA and therefore differed between the years.

Figure 4 shows the number of participating laboratories for all three EQAs and their penalty points, respectively. Over time, the number of participating laboratories decreased. Roughly two-thirds of laboratories that participated in the first EQA also participated in the second EQA. A total of 27 laboratories from 25 countries participated in all three EQAs. Of these, 19 scored less than 100% in 2020. In 2021, 10 laboratories scored less than 100% and in 2023, only 4 laboratories scored below 100%. The evaluation of the individual laboratories over time can be seen in Fig. 4.

## DISCUSSION

We hereby report the results of the third European EQA of molecular detection of SARS-CoV-2 and the second that included variant identification, conducted in 2023. Compared to the two previous EQAs (1, 2), fewer laboratories participated. In the first EQA, all EVD-Labnet laboratories as well as influenza contact points were invited. In the course of the pandemic, operational contact points for SARS-CoV-2 were identified. These were the institutes that were invited for the current EQA. Given only a slight decrease in the number of countries represented, now typically only one laboratory per country participated. All laboratories participating in this EQA also participated in the first and/or second EQAs.

Compared with former EQAs, an improvement in performance over time could be detected, starting with 12% of participating laboratories who classified all SARS-CoV-2 containing samples correctly as SARS-CoV-2 RNA positive in 2021 and 75% in 2022. In 2023, 90% of the participating laboratories correctly classified all SARS-CoV-2 containing samples. As expected, reduced molecular detection levels (95%) were only observed for SARS-CoV-2 containing samples with low RNA concentrations (≤13 cps/µL). This was already observed in previous EQAs (1, 2). Here, samples with comparable concentrations (11 and 12.5 cps/µL) were identified correctly by 91.5% and 90–99% of participants (1, 2). However, a lower diagnostic sensitivity for samples with such low SARS-CoV-2 RNA concertation is of limited clinical importance (8, 9). Although higher SARS-CoV-2 RNA concentration showed a lower ct value, it is, however, no quantitative measure. Different laboratories used different assays leading to different ct values for the same sample. To be able to compare the results of different laboratories, truly comparable measures like concentration of viral RNA based on standardized positive controls are indispensable. This fact demonstrates the urgent need for reference material, enabling the comparison between laboratories and quantitative statements.

One laboratory participating in the current EQA reported three SARS-CoV-2 containing samples as false negatives. This laboratory used an in-house assay and classified samples as negative if their ct value was above 42. Two laboratories identified four SARS-CoV-2 containing samples in total as inconclusive with ct values between 36 and 38. Of these laboratories, 50% used an in-house assay and 50% used a commercial kit. The commercial kit was also used by other participating laboratories correctly detecting all SARS-CoV-2-containing samples. Therefore, false negative or inconclusive results were most probably due to extraction procedures and/or pre-RT-PCR workflow. Two laboratories that reported false positive results, both used commercial kits that were also used error-free by other laboratories. As a result, false-positive test results were more likely a consequence of contamination during sample extraction, handling, admin error, or lot-specific contamination of rRT-PCR (real-time reverse transcription PCR) kits or oligonucleotides (1, 2) rather than non-specificity of the RT-PCR. In general, 27 laboratories used eleven different commercial kits. In the current EQA, variant typing of the SARS-CoV-2 positive samples was voluntary, and 34% (13/38) of participants performed typing. Unlike the detection aspect of the EQA, the typing results were quite diverse. Approximately half of the laboratories correctly identified most of the specific SARS-CoV-2 variants. Incorrect or no results were primarily reported for samples with low viral RNA concentrations. As viral load is a major factor influencing sequencing success (10), and low RNA concentrations can both lead to sequencing errors and increase the risk of general test failure (11), these outcomes were as expected. For future EQAs, the design of the samples and their viral load should be reconsidered. Nevertheless, these results indicate a performance gap between the labs especially for low viral content specimens that may need to be addressed, especially given the increasing importance of molecular surveillance of pathogens (12, 13). Genomic pathogen surveillance enables real-time tracking of infection spread across populations. Additionally, it allows for the detection of changes in the pathogen, and presents information that can be used to adjust measures to control a pandemic (14). The results of this EQA were discussed during the ECDC WHO Europe Laboratory Network meeting along with available training opportunities.

To specifically address the issues with variant typing repeated drylab EQAs could be performed. As demonstrated by a pilot EQA focusing on SARS-CoV-2 whole-genome sequencing that was published in early 2022, most participating laboratories generated whole-genome sequences and correctly identified all lineages and clusters; however, coverage across the genome was the most critical aspect, correlating with correct lineage and cluster assignments. Sequencing depths varied by as much as 100-fold and consistent with our EQA findings, many labs in the drylab EQA struggled with a sample that had a lower viral load (ct 28). The main causes of these inconsistencies were missing data, bioinformatic choices, and data interpretation (10).

Our results from three European SARS-CoV-2 EQAs suggest that repeated EQAs can contribute to improved detection capabilities of laboratories. We also observed a shift from in-house tests to more commercial tests and automated extraction systems, which aligns with the establishment of SARS-CoV-2 as an endemic pathogen. This trend might be strengthened by the European IVDR, which favors commercially available CE IVD products for diagnostics. However, such kits are often not available for rare or emerging viruses, nor during the initial phase of a pandemic.

To further improve SARS-CoV-2 genomic surveillance and variant typing, future EQAs should focus on sequencing, as part of wet-lab EQAs shown here, but also additionally via dedicated dry-lab exercises. Increasing the sensitivity of sequencing protocols and the correct interpretation of the sequencing data are crucial for proper variant typing.

## SUPPLEMENTAL MATERIAL

Figure S1 jcm.01538-24-s0001.docx Map of participating laboratories by country, European SARS-CoV-2 EQA, 2023.

ASM does not own the copyrights to Supplemental Material that may be linked to, or accessed through, an article. The authors have granted ASM a non-exclusive, world-wide license to publish the Supplemental Material files. Please contact the corresponding author directly for reuse.
