# Supplementary material for: External quality assessment of molecular detection and variant typing of SARS-CoV-2 in European expert laboratories in 2023
Source: J Clin Microbiol. 2025 Mar 14;63(4):e01538-24. doi: 10.1128/jcm.01538-24 (PMC11980390; doi:10.1128/jcm.01538-24)
Supplement: Figure S1 — Map of participating laboratories by country, European SARS-CoV-2 EQA, 2023. [file jcm.01538-24-s0001.docx]

**Supplemental material**


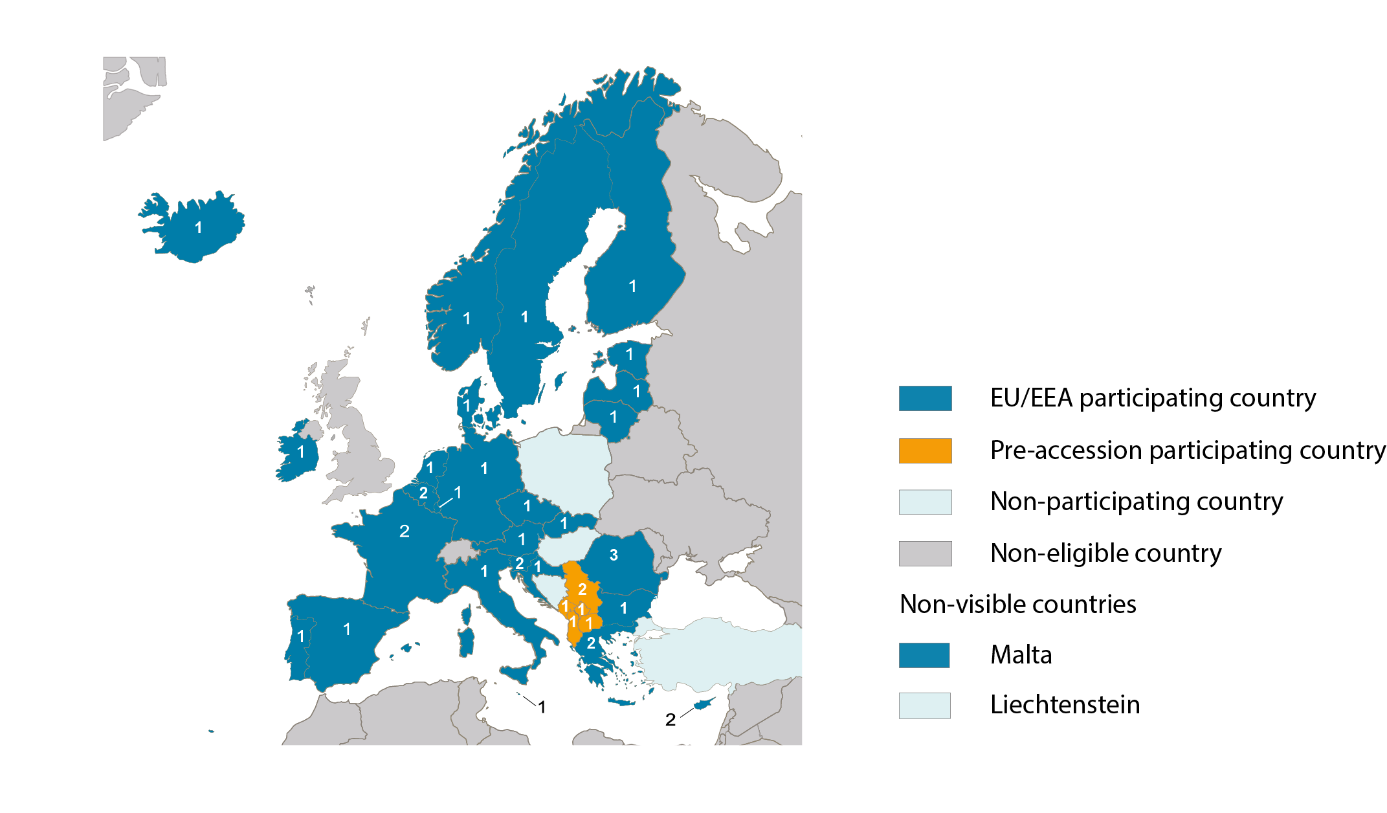


***Figure S1. Map of participating laboratories by country, European SARS-CoV-2 EQA, 2023. The number of participating laboratories per country is shown on the map.***
